# Big Data, Decision Models, and Public Health

**DOI:** 10.3390/ijerph17186723

**Published:** 2020-09-15

**Authors:** Chien-Lung Chan, Chi-Chang Chang

**Affiliations:** 1 Department of Information Management, Yuan Ze University, Taoyuan 320, Taiwan; clchan@saturn.yzu.edu.tw; 2 Innovation Center for Big Data and Digital Convergence, Yuan Ze University, Taoyuan 320, Taiwan; 3 School of Medical Informatics, Chung Shan Medical University & IT Office, Chung Shan Medical University Hospital, Taichung 40201, Taiwan

**Keywords:** big data, decision models, public health

## Abstract

Unlike most daily decisions, medical decision making often has substantial consequences and trade-offs. Recently, big data analytics techniques such as statistical analysis, data mining, machine learning and deep learning can be applied to construct innovative decision models. With complex decision making, it can be difficult to comprehend and compare the benefits and risks of all available options to make a decision. For these reasons, this Special Issue focuses on the use of big data analytics and forms of public health decision making based on the decision model, spanning from theory to practice. A total of 64 submissions were carefully blind peer reviewed by at least two referees and, finally, 23 papers were selected for this Special Issue.

## 1. Introduction

In the digital era, the volume and velocity of environmental, population and public health data from a diverse range of sources are growing rapidly. Big data analytics techniques such as statistical analysis, data mining, machine learning and deep learning, etc., have made great progress in recent decades and attracted the growing interest of researchers and scientists in a number of various applications. In particular, decision making based on concrete evidence is critical and has a substantial impact on public health and program implementation. While working with people’s health and medical information, we also need to commit to scientific integrity issues including people’s privacy, data sharing, bias and uncertainty, research design and statistical inference. For these reasons, this Special Issue focuses on the use of big data analytics and forms of public health decision making based on the decision model, spanning from theory to practice. Our Special Issue received 64 high-quality submissions. All the submitted manuscripts were carefully blind peer reviewed by at least two referees, in accordance with *International Journal of Environmental Research and Public Health* (IJERPH) review procedure and, finally, 23 papers were selected for this Special Issue. This Special Issue has successfully addressed the critical research needs for Big Data, Decision Models, and Public Health. The guest editors would like to express their sincere appreciations to all reviewers for their invaluable contributions in reviewing the manuscripts and providing constructive feedback. The guest editors also sincerely thank *International Journal of Environmental Research and Public Health* for offering this great opportunity to organize this Special Issue.

## 2. The Organization of This Special Issue

This Special Issue essentially covers five important themes. The first theme looks at the preventive medicine and risk assessment. The second theme looks at the forecasting models to support healthcare policies. The third theme considers big data analytics for improving public health and chronic diseases. The fourth theme investigates the potential risks and benefit that are associated with the disease control. Finally, the fifth theme describes the health-related quality of life.

### 2.1. Preventive Medicine and Risk Assessment

Preventive care is the most important issue of healthcare activities. The developmental origin of the health and disease (DOHaD) concept proposes that prenatal and early postnatal exposures increase disease susceptibility throughout life. The first paper, “*Chained Risk Assessment for Life-Long Disease Burden of Early Exposures–Demonstration of Concept Using Prenatal Maternal Smoking*”, by Rumrich et al. [1], examines the application of the DOHaD concept in a chained risk assessment and to provide an estimate of later in life burden of disease related to maternal smoking. The results confirm the potential to explain a previously unattributed part of the non-communicable diseases by the DOHaD concept. It is likely that relevant outcomes are missing, resulting in an underestimation of disease burden.

The identification and monitoring of occupational cancer are important aspects of occupational health protection. The second paper, “*Estimation of Occupational Exposure to Asbestos in Italy by the Linkage of Mesothelioma Registry (ReNaM) and National Insurance Archives. Methodology and Results*”, by Airoldi et al. [2], creates a list of industries with asbestos exposure and identifies cancer cases of who worked in these industries. Eighteen percent of the cancer cases showed at least one work period in firms potentially exposed to asbestos, 48% of which in regions different from where the cases lived at diagnosis. The methodology offers support for the preliminary screening of asbestos-exposed firms in the occupational history of cancer cases.

The third paper, “*The Association between Metabolically Healthy Obesity, Cardiovascular Disease, and All-Cause Mortality Risk in Asia: A Systematic Review and Meta-Analysis*”, by Huang et al. [3], investigates the association among metabolically healthy obesity (MHO), cardiovascular disease (CVD) risk, and all-cause mortality in the Asian population. Participants with MHO had a significantly lower risk of all-cause mortality than MHNO (non-obese, including overweight and underweight) participants, but a borderline significantly higher risk of all-cause mortality than MHNW (without overweight or underweight) participants. Particularly, the CVD risk and all-cause mortality of the MHO group changed depending on the control group; therefore, future studies should select control groups carefully.

The fourth paper, “*APOE Variant (rs405509) might Modulate the Effect of Sex and Educational Level on Cognitive Impairment Risk in a Taiwanese Population*”, by Hsu et al. [4], investigates if the APOE-rs405509 genotypes (TT, TG, and GG) modulate the effect of sex and education on cognitive impairment in Taiwanese adults. After stratification by rs405509 genotypes, this association was significant only among TT genotype carriers. A significant association between MMSE score and sex was observed in the lowest educational group, especially among carriers of rs405509 TT genotypes.

### 2.2. Forecasting Models to Support Healthcare Policies

This theme looks at the health services and support to decision makers in implementing targeted health policies more efficiently. The first paper, “*Time Series Analysis and Forecasting with Automated Machine Learning on a National ICD-10 Database*”, by Olsavszky et al. [5], deployed a novel Machine Learning, called automated time series (AutoTS) machine learning, to automate data processing and the application of a multitude of models to assess which best forecasts future values. By using the nation-wide ICD-10 (International Classification of Diseases, Tenth Revision) dataset of hospitalized patients of Romania, authors have generated time series datasets over the period of 2008–2018 and performed highly accurate AutoTS predictions for the 10 deadliest diseases. Especially, the deployment of AutoTS technology can help decision makers in implementing targeted national health policies more efficiently.

Against a rapidly aging population, projections are done to size up the demand for long-term care (LTC) services for long-range policy planning. The second paper, “*40-Year Projections of Disability and Social Isolation of Older Adults for Long-Range Policy Planning in Singapore*”, by Ng et al. [6], completes a 40-year projection of LTC demand based on disability and social isolation in Singapore. Regression models of living arrangements revealed interesting ethnic differences: Malay elders are 2.6 times less likely to live alone than their Chinese counterparts, controlling for marital status, age, and housing type. These projections provide a glimpse of the growing demand for LTC services for a rapidly aging Singapore and underscore the need to shore up community-based resources to enable seniors to age in place.

With the rapid development of the COVID-19 pandemic, countries are trying to cope with increasing medical demands, and, at the same time, to reduce the increase of infected numbers by implementing a number of public health measures, namely non-pharmaceutical interventions (NPIs). The third paper, “*The Outcome and Implications of Public Precautionary Measures in Taiwan–Declining Respiratory Disease Cases in the COVID-19 Pandemic*”, by Hsieh et al. [7], presents evidence that spread prevention involving mass masking and universal hygiene at the early stage of the COVID-19 pandemic resulted in a 50% decline of infectious respiratory diseases, based on historical data during the influenza season in Taiwan. These outcomes provide potential support for the effectiveness of widely implementing public health precaution measures in controlling COVID-19 without a lockdown policy.

Influenza is a serious public health issue, as it can cause acute suffering and even death, social disruption, and economic loss. Effective forecasting of influenza outpatient visits is beneficial to anticipate and prevent medical resource shortages. The fourth paper, “*Forecasting Weekly Influenza Outpatient Visits Using a Two-Dimensional Hierarchical Decision Tree Scheme*”, by Lee et al. [8], used regional data on influenza outpatient visits to propose a two-dimensional hierarchical decision tree scheme for forecasting influenza outpatient visits. The results suggest that, for forecasting nationwide influenza outpatient visits in Taiwan, one- and two-time lag information and regional information from the Taipei, North, and South regions are significant.

The fifth paper, “*A Population-Based Study of Healthcare Resource Utilization in Patients with Mitral Valve Prolapse*”, by Chen et al. [9], investigates differences in the utilization of healthcare services between subjects with mitral valve prolapse (MVP). Multiple regression analysis indicated that patients with MVP had higher total costs for all healthcare services than patients without MVP after adjusting for the urbanization level, monthly income, and geographic region. This study demonstrated that healthcare utilization by patients with MVP is substantially higher than comparison patients.

### 2.3. Big Data Analytics for Improving Public Health and Chronic Diseases

This theme considers big data analytics for improving public health and chronic diseases. The first paper, “*Bone Mineral Density of Femur and Lumbar and the Relation between Fat Mass and Lean Mass of Adolescents: Based on Korea National Health and Nutrition Examination Survey (KNHNES) from 2008 to 2011*”, by Kim et al. [10], identifies the relationship between bone density and both fat mass and lean mass of Korean adolescents. Subjects were chosen among 21,303 people from the Korea National Health and Nutrition Examination Survey (KNHNES) between 2008 and 2011 that took a bone density checkup. For male adolescents, the bone density differences for fat mass (FM) and lean mass (LM) all had significant differences, but for female adolescents, only the lumbar spine for LM showed such a result. Meanwhile, both genders showed that LM had a more positive impact on bone density than FM.

The second paper, “*Hyperlipidemia and Statins Use for the Risk of New Diagnosed Sarcopenia in Patients with Chronic Kidney: A Population-Based Study*”, by Lin et al. [11], assessed the risk for new-onset sarcopenia among patients with chronic kidney disease using statins. In a nationwide retrospective population-based cohort study, 75,637 clinically confirmed cases of chronic kidney disease between 1997 and 2011 were selected from the National Health Insurance Research Database (NHIRD) of Taiwan. Patients with chronic kidney disease could receive statin treatment to reduce the occurrence of newly diagnosed sarcopenia. Additionally, a higher dosage of statins could reduce the incidence of newly diagnosed sarcopenia in patients with chronic kidney disease.

The third paper, “*The Risk of Depression in Patients with Pemphigus: A Nationwide Cohort Study in Taiwan*”, by Hsu et al. [12], investigates the risk of depression in patients with pemphigus. Data were derived from the NHIRD recorded during the period 2000–2010 in Taiwan. Multivariate Cox proportional hazards regression models were used to analyze the data and assess the effects of pemphigus on the risk of depression after adjusting for demographic characteristics and comorbidities. There was a significant association between pemphigus and increased risk of depression. Female patients had a higher incidence of depression. People with HTN, hyperlipidemia, asthma/COPD, and chronic liver disease were, respectively, 1.73, 2.3, 2.2, and 1.69 times more likely to suffer from depression than those without these comorbidities.

Developing effective risk prediction models is a cost-effective approach to predicting complications of chronic kidney disease (CKD) and mortality rates; however, there is inadequate evidence to support screening for CKD. The fourth paper, “*Risk Prediction for Early Chronic Kidney Disease: Results from an Adult Health Examination Program of 19,270 Individuals*”, by Shih et al. [13], proposes four data-mining algorithms that are used to predict early CKD. The study includes datasets from 19,270 patients, provided by an adult health examination program between 2015 and 2019. The experimental results showed that Urine protein and creatinine ratio (UPCR), Proteinuria (PRO), Red blood cells (RBC), Glucose Fasting (GLU), Triglycerides (TG), Total Cholesterol (T-CHO), age, and gender are important risk factors. CKD care is closely related to primary care level and is recognized as a healthcare priority in national strategy. Finally, the proposed risk prediction models can also support the important influence of personality and health examination representations in predicting early CKD.

Recurrence of paroxysmal supraventricular tachycardia (PSVT) has been reported to be lower in patients treated with radiofrequency catheter ablation (RFCA) than in those who are not. Few population-based surveys have stated the cost-effectiveness related to this treatment. The fifth paper, “*Cost Effectiveness Analysis and Payment Policy Recommendation—Population-Based Survey with Big Data Methodology for Readmission Prevention of Patients with Paroxysmal Supraventricular Tachycardia treated with Radiofrequency Catheter Ablation*”, by Chan et al. [14], performed a nationwide retrospective study using NHIRD data from 2001–2012 in Taiwan. There were 21,086 patients hospitalized due to first-time PSVT, of whom 13,075 underwent RFCA, with 374 recurrences (2.86%). The PSVT recurrence rate was much higher in patients who did not receive RFCA at their first admission. RFCA proved cost-effective, with the ratio of the incremental cost-effectiveness ratio (ICER) and gross domestic product (GDP) being only 1.15. To prevent readmission and avoid incremental cost, the authority could provide a financial supplement for every patient so that the procedure is performed, reducing the PSVT recurrence life years (disease-specific DALY).

Most stroke cases lead to serious mental and physical disabilities, such as dementia and sensory impairment. Chronic diseases are contributory risk factors for stroke. However, few studies considered the transition behaviors of stroke to dementia associated with chronic diseases and environmental risks. The sixth paper, “*Stroke to Dementia Associated with Environmental Risks—A Semi-Markov Model*”, by Wang et al. [15], developed a prognosis model to address the issue of stroke transitioning to dementia associated with environmental risks. Multivariate analysis showed that certain environmental risks, medication, and rehabilitation factors highly influenced the transition of stroke from a chronic disease to dementia. Finally, the proposed model also facilitated an accurate prognosis on the transition time of stroke from chronic diseases to dementias against environmental risks and rehabilitation factors.

### 2.4. The Disease Control and Treatment Outcome

This theme investigates the potential risks and benefits that are associated with disease control. Ischemic stroke is the most common type of stroke, and early interventional treatment is associated with favorable outcomes. In the guidelines, thrombolytic therapy using recombinant tissue-type plasminogen activator (rt-PA) is recommended for eligible patients with acute ischemic stroke. However, the risk of hemorrhagic complications limits the use of rt-PA, and the risk factors for poor treatment outcomes need to be identified. The first paper, “*Factors Associated with Outcomes of Recombinant Tissue Plasminogen Activator Therapy in Patients with Acute Ischemic Stroke*”, by Tseng et al. [16], analyzes the electronic medical records of patients who were diagnosed with acute ischemic stroke and treated for rt-PA. In the multivariable analysis, risk factors associated with poor outcomes were female gender, higher stroke severity index (SSI), higher serum glucose levels, lower mean corpuscular hemoglobin concentration (MCHC), lower platelet counts, and anemia. The risk factors found in this research could help us study the treatment strategy for ischemic stroke.

The second paper, “*Interleukin-3 Polymorphism is Associated with Miscarriage of Fresh in Vitro Fertilization Cycles*”, by Wu et al. [17], examines the association between interleukin (IL) genes polymorphisms and in vitro fertilization (IVF) outcome. The main outcome measures included clinical pregnancy, embryo implantation, abortion and live birth rates. Infertile women with IL-3 homozygous major genotype had a higher abortion rate than those with heterozygous and homozygous minor genotype. In their conclusion, IL-3 rs40401 polymorphism is associated with increased risk of abortion of IVF patients.

The third paper, “*Increased One-Year Recurrent Ischemic Stroke after First-Ever Ischemic Stroke in Males with Benign Prostatic Hyperplasia*”, by Cheng et al. [18], determines the risk of one-year recurrent Ischemic Stroke (IS) conferred by Benign Prostatic Hyperplasia (BPH). They found that patients with BPH had a higher risk of recurrent IS. Other risk factors included hyperlipidemia, coronary artery disease, chronic obstructive pulmonary disease, and chronic kidney disease. Patients with BPH who had these risk factors had an increased risk of one-year recurrent IS. Possibly, the modification of risk factors may prevent recurrent IS.

Non-genotoxic hepatocarcinogens (NGHCs) can only be confirmed by 2-year rodent studies. Toxicogenomics (TGx) approaches using gene expression profiles from short-term animal studies could enable early assessment of NGHCs. However, high variance in the modulation of the genes had been noted among exposure styles and datasets. The fourth paper, “*Identification of Time-Invariant Biomarkers for Non-Genotoxic Hepatocarcinogen Assessment*”, by Huang et al. [19], identifies time-invariant biomarkers for NGHCs in short-term exposure styles and validate their applicability to long-term exposure styles. Machine learning techniques were subsequently employed to assess the prediction performance of the biomarkers. In addition, enrichment analysis of the biomarkers inferred the involvement of chronic inflammatory diseases such as liver cirrhosis, fibrosis, and hepatocellular carcinoma in NGHCs. Finally, the time-invariant biomarkers provided a robust alternative for NGHC prediction.

The link between diabetes and hypertension is mutual and reciprocal, increasing the risks for the development of atrial fibrillation (AF). The fifth paper, “*Incidence and Risk Assessment for Atrial Fibrillation at 5 Years: Hypertensive Diabetic Cohort*”, by Muria-Subirats et al. [20], develops a prediction model for AF in a population with both diabetes and hypertension at five years of follow-up. Multivariate Cox proportional-hazards regression models were used to identify predictors AF and to stratify risk scores by quartiles. They found that risk-based screening for AF should be used in high-cardiovascular-risk patients as the hypertensive diabetics, for treatment of modifiable cardiovascular risk, and monitoring AF detection.

### 2.5. Health-Related Quality of Life

This theme describes the health-related quality of life. Patients with either osteoporosis or depression are prone to develop other diseases and require more medical resources than the general population. However, there are no studies on health-related quality of life (HRQoL) and medical resource use by osteoporosis patients with comorbid depression. The first paper, “*Health-Related Quality of Life and Medical Resource Use in Patients with Osteoporosis and Depression: A Cross-Sectional Analysis from the National Health and Nutrition Examination Survey*”, by Weng et al. [21], uses multivariate linear and logistic regression model to analyze the HRQoL and medical resource use between groups. Each patient was assigned to one of four groups: osteoporosis-positive^(+)^ and depression-positive^(+)^ (O^+^/D^+^); O^+^/D^−^; O^−^/D^+^; O^−^/D^−^. Low HRQoL was significantly more prevalent in O^+^/D^+^ patients. Authors found that depression severity more significantly affected HRQoL than did osteoporosis. However, both diseases significantly increased the risk of high medical resource use.

Previous studies have proposed various physical tests for screening fall risk in older adults. However, older adults may have physical or cognitive impairments that make testing difficult. The second paper, “*Physical and Psychological Factors Associated with Poor Self-Reported Health Status in Older Adults with Falls*”, by Kim et al. [22], describes the differences in individual, physical, and psychological factors between adults in good and poor self-rated health statuses. Multivariable logistic regression revealed that poor self-reported health was significantly associated with hearing impairments, activities of daily living (ADL) limitation, instrumental activities of daily living (IADL) limitation, poor nutrition, and depression. They found that auditory impairment, ADL/IADL limitations, poor nutrition, and depression were significantly associated with poor self-reported health. A self-rated health assessment could be an alternative tool for older adults who are not able to perform physical tests.

The autonomic dysfunction in palmar hyperhidrosis (PH) includes not only sympathetic overactivity but also parasympathetic impairment. A decrease of parasympathetic tone has been noted in gastroesophageal reflux disease of neonates and adults. The third paper, “*Association between Reflux Esophagitis Incidence and Palmar Hyperhidrosis*”, by Cheng et al. [23], deliberates the association between reflux esophagitis and PH. The risk of reflux esophagitis in PH patients had a hazard ratio of 3.457 after adjustment of the other factors. It confirmed the association between reflux esophagitis and PH. Health care providers must be alerted to this relationship and other risk factors of reflux esophagitis to support suitable treatments to improve the quality of life of patients.
